# Intravascular Ultrasound and Angiographic Predictors of In-Stent Restenosis of Chronic Total Occlusion Lesions

**DOI:** 10.1371/journal.pone.0140421

**Published:** 2015-10-14

**Authors:** Jeehoon Kang, Young-Seok Cho, Seong-Wook Kim, Jin Joo Park, Yeonyee E. Yoon, Il-Young Oh, Chang-Hwan Yoon, Jung-Won Suh, Tae-Jin Youn, In-Ho Chae, Dong-Ju Choi

**Affiliations:** 1 Department of Internal Medicine, Seoul National University Bundang Hospital, Seongnam, South Korea; 2 Department of Internal Medicine, Seoul National University Hospital, Seoul, South Korea; 3 Department of Internal Medicine, Seoul National University College of Medicine, Seoul, South Korea; Federico II University, Naples, ITALY

## Abstract

Despite the benefits of successful percutaneous coronary interventions (PCIs) for chronic total occlusion (CTO) lesions, PCIs of CTO lesions still carry a high rate of adverse events, including in-stent restenosis (ISR). Because previous reports have not specifically investigated the intravascular ultrasound (IVUS) predictors of ISR in CTO lesions, we focused on these predictors. We included 126 patients who underwent successful PCIs, using drug-eluting stents, and post-PCI IVUS of CTO lesions. Patient and lesion characteristics were analyzed to elucidate the ISR predictors. In each lesion, an average of 1.7 ± 0.7 (mean length, 46.4 ± 20.3 mm) stents were used. At 9 months follow-up, 14 (11%) patients demonstrated ISR, and 8 (6.3%) underwent target lesion revascularization. Multivariate logistic regression analysis showed that the independent predictors of ISR were the post-PCI minimal luminal diameter (MLD) and the stent expansion ratio (SER; minimal stent cross-sectional area (CSA) over the nominal CSA of the implanted stent), measured using quantitative coronary angiography (QCA) and IVUS, respectively. A receiver operating characteristic analysis indicated that the best post-PCI MLD and SER cut-off values for predicting ISR were 2.4 mm (area under the curve [AUC], 0.762; 95% confidence interval (CI), 0.639–0.885) and 70% (AUC, 0.714; 95% CI, 0.577–0.852), respectively. Lesions with post-PCI MLD and SER values less than these threshold values were at a higher risk of ISR, with an odds ratio of 23.3 (95% CI, 2.74–198.08), compared with lesions having larger MLD and SER values. Thus, the potential predictors of ISR, after PCI of CTO lesions, are the post-PCI MLD and SER values. The ISR rate was highest in lesions with a post-PCI MLD ≤2.4 mm and an SER ≤70%.

## Introduction

As percutaneous coronary intervention (PCI) techniques and skills have improved, chronic total occlusions (CTO) have become important targets for percutaneous revascularization. The benefits of PCI for CTO lesions include symptomatic relief, improved left ventricular function, and enhanced survival [1]. Despite these potential benefits, PCI of CTO lesions is difficult owing to its procedural complexity [2], and has a relatively low success rate, with a relatively high rate of in-stent restenosis (ISR) [3, 4].

ISR, induced by neointimal hyperplasia, is a long-recognized, chronic complication following PCI. In particular, the ISR rate after PCI of CTO lesions is well-known to be higher than that associated with standard stenotic coronary lesions [4, 5]. According to several studies, CTO lesions have a 1.4- to 5-fold higher rate of ISR than standard coronary lesions [5–10]. Due to this high ISR rate, the identification of clinical and/or angiographic characteristics that predict ISR is both essential and clinically important.

The intravascular ultrasound (IVUS) predictors of ISR of CTO lesions have not been studied, unlike those for non-CTO lesions. CTO lesions are distinct from other lesions owing to the presence of large plaque burdens, greater lesion lengths, frequent severe calcification, and shrunken distal reference vessels [11]. These distinct characteristics justify a specific study of ISR in CTO lesions. In this study, by evaluating CTO lesions in the post-PCI period, we sought to identify the predictors of ISR. Additionally, we utilized IVUS as a tool to evaluate plaque characteristics (i.e., lumen area, vessel area, and plaque burden) and to obtain in-depth analyses of the lesions.

## Methods

### Study population

This was an exploratory study involving a retrospective analysis. The protocol was approved by the Ethics Committee and Institutional Review Board at Seoul National University Bundang Hospital and was conducted according to the principles of the Declaration of Helsinki. Due to the retrospective nature of the study, the need for verbal or written consent was waived by the Ethics Committee and Institutional Review Board of Seoul National University Bundang Hospital. Seoul National University Bundang Hospital patients who underwent PCIs for CTO lesions, between January 2006 and December 2013, and who also participated in angiographic follow-up evaluations were included in this study. We have a standardized CTO intervention protocol that involves the routine use of IVUS to increase procedural success and to minimize procedure-related complications. The protocol also includes angiographic follow-up to assess the patency of the recanalized vessels. Therefore, most CTO patients experiencing successful recanalizations were included. Patients were excluded if they had allergies to study related medications (antiplatelet drugs, heparin, metal alloys, or contrast agents), had a planned surgery within 6 months of PCI or had planned thrombolysis, were pregnant, were <18-years-old or >95-years-old, had angina not due to coronary disease, or had a life expectancy of <6 months.

A power analysis of our study sample was conducted as follows. We used a combination of two ISR predictors, the post-PCI Minimal luminal diameter (MLD) and the Stent expansion ratio (SER). When using single predictors and an α (type I error rate) value of 0.05, the power of the study was 72% and 92% (for the post-PCI MLD and SER, respectively). However, after combining the predictors, the power improved to 99%.

**Fig 1:**
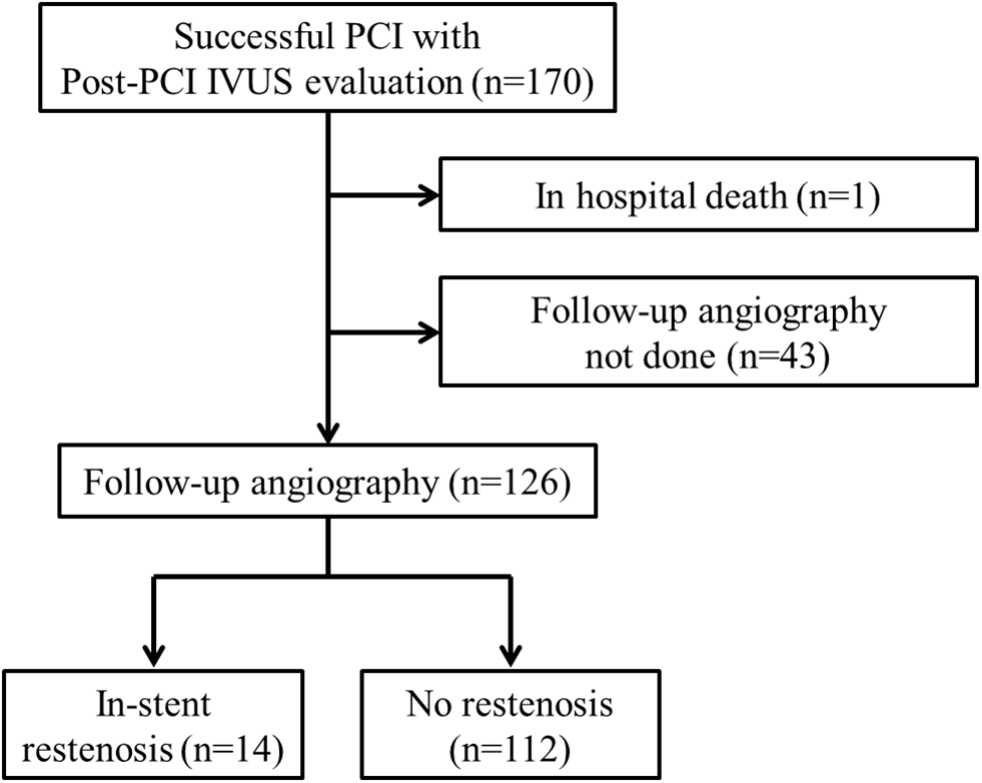
Study profile of the patients.

A total of 170 patients underwent successful PCIs of CTO lesions and completed post-PCI IVUS evaluations. Out of this population, one patient died during the initial hospitalization and 43 patients did not receive follow-up coronary angiography. Among those who did not receive follow-up coronary angiography, 30 patients were followed-up in the outpatient clinic, but refused angiography due to financial reasons (22 patients) or inflexible work hours (8 patients). Further, 12 patients were lost to follow-up, and 1 patient died as a result of an intracranial hemorrhage 4 months after PCI. Therefore, successful 9-month follow-up coronary angiography was performed in 126 patients (74%) (Fig 1). Patients who did not receive 9-month follow-up coronary angiography were not significantly different from those who received coronary angiography, except for being older (S1 Table).

### PCI procedures

PCI was performed using standard techniques and the femoral approach, with the specific revascularization technique left to the surgeon’s discretion. CTO recanalization was performed via an antegrade approach in 121 (96.0%) patients and via a retrograde approach in 5 (4.0%) patients. Among the 121 patients undergoing the antegrade approach, a single-wire drilling or penetrating technique was used in 98 patients; a parallel-wire technique was used in 21, and a subintimal tracking and re-entry technique in 2. Among the 5 patients undergoing the retrograde approach, 4 underwent the single-wire drilling or penetrating technique and 1 underwent the reverse controlled antegrade and retrograde sub-intimal tracking technique.

Drug-eluting stents were used in all cases, and successful PCI was defined as a minimum diameter stenosis of <10%; a final thrombolysis in myocardial infarction flow grade of 3; and the absence of significant side branch occlusion, flow-limiting dissection, distal embolization, or angiographic thrombus [12]. Each patient underwent a final IVUS evaluation, after stent implantation and adjunctive balloon dilatation.

All patients received a loading dose of aspirin (300 mg) and clopidogrel (300–600 mg) before the PCI. Following PCI, patients were recommended to continue clopidogrel (75 mg/day) for at least 1 year and to continue aspirin (100 mg/day) indefinitely. Other medications, such as beta-blockers or nitrates, were administered at the discretion of the attending physician. Nine-month follow-up coronary angiography was offered to all patients, regardless of whether or not it was clinically indicated at an earlier time point. Three patients underwent angiography at an earlier time point, and each also underwent the 9-month follow-up angiogram.

### Angiographic and IVUS analyses

Quantitative coronary angiography (QCA) was performed using standard techniques with automated edge-detection algorithms (CAAS 5.7.1, Pie Medical Imaging, Masstricht, The Netherlands) in the hospital’s angiographic analysis center. ISR was diagnosed if the diameter stenosis was >50% of the stented segment at the follow-up coronary angiography [13].

IVUS (Volcano, Rancho Cordova, CA, USA and Boston Scientific/SCIMED, Minneapolis, MN, USA), consisting of a rotating 20-MHz (Eagle Eye^®^ Platinum Catheter, Volcano) or 40-MHz transducer (Atlantis^TM^ SR Pro Imaging Catheter, Boston Scientific), analysis was performed before stenting (post-ballooning) in 37 (29.4%) patients, and immediately after stenting in 89 (70.6%); each patient underwent additional final IVUS imaging next to the end of CTO revascularization. Final post-stenting IVUS imaging was performed after the intracoronary administration of nitroglycerin (0.2 mg) using a motorized transducer (0.5 mm/s). However, follow-up IVUS imaging was not performed unless clinically necessary. The minimal stent area (MSA) was the smallest cross-sectional area (CSA) measured within the stented segment; the SER was defined as the ‘MSA divided by the nominal CSA of the implanted stent’. At the MSA site, the CSA of the external elastic membrane area (EEMA) [14] was also measured. ‘behind-stent plaque CSA’ was calculated as EEMA minus MSA and ‘percentage area stenosis’ was calculated as (plaque area/EEMA) × 100.

### Inter- and intra-observer analysis

To guarantee the quality and reproducibility of the analytical measurements, we performed an inter- and intra-observer analysis. For representative analysis values, the ‘pre-PCI reference diameter’, ‘post-PCI reference diameter’, ‘post-PCI MLD’, ‘follow-up reference diameter’, and ‘follow-up MLD’ were used to analyze QCA variables; the ‘MSA’ and ‘EEMA at the MSA site’ measurements were used in the IVUS analysis. For inter-observer variability, both the QCA and IVUS analyses were each performed by two experienced observers. For intra-observer variability, one investigator analyzed the representative variables twice, after an interval of no longer than 5 min. The agreement of the results was analyzed using the Bland–Altman plot and intraclass correlation coefficient determination (S2–S5 Tables, S1 and S2 Figs).

### Statistical analysis

Data are presented as numbers and frequencies for categorical variables and as means ± SD for continuous variables. To compare groups, the χ^2^ test (or Fisher’s exact test, when any expected cell count was <5 for a 2 × 2 table) was used for categorical variables, and an unpaired Student’s *t*-test or one-way analysis of variance was applied for continuous variables.

For the multiple regression analysis used to identify variables influencing ISR, a binary logistic model, based on multiple predictor variables, was used. Candidate variables with a p <0.10 in the univariate analyses and known ISR risk factors (i.e., calcification and diabetes mellitus) were included in the model. Assumptions of the logistic regression model (e.g., dichotomous dependent variable, independence of each observation, linear relationship between continuous independent variables and logit transformations of the dependent variables, and multi-collinearity) were checked. The performances of variables included in the logistic regression model were checked using the Akaike information criterion and Bayesian information criterion values. The Cox and Snell R-Square, Nagelkerke R-square, and Hosmer-Lemeshow goodness-of-fit tests were used to evaluate model calibration. The final model was determined using a backward variable selection approach. To predict ISR, a receiver operating curve was used.

All statistical tests were two-tailed and a two-sided probability value <0.05 was considered statistically significant. Statistical tests were performed using SPSS, version 18 (SPSS, Chicago, IL, USA) and STATA, version 10 (STATA, College Station, TX, USA).

## Results

### Baseline characteristics and 9-month follow-up

The baseline clinical characteristics are shown in Table 1. Of the total population, 36.5% of the patients were diagnosed with acute coronary syndrome; 2 had ST-segment elevation myocardial infarctions and 8 had non–ST-segment elevation myocardial infarctions. The CTO lesion was the non-culprit lesion in all acute myocardial infarction (AMI) patients. The average follow-up duration from the index PCI to the follow-up angiogram was 241 ± 83 d. Follow-up coronary angiography showed ISR of the previously revascularized CTO lesion in 14 (11.1%) patients (Fig 1); 7 were focal, 1 was diffuse and intrastent, and 6 showed total occlusion. Among the 7 patients with focal ISR, 2 had edge ISR (one each with proximal-edge and distal-edge ISR). During the follow-up period, 8 (6.3%) patients showed target lesion revascularization and 9 (7.1%) showed target vessel revascularization; there were no cases of myocardial infarction or stent thrombosis. There were no significant differences in baseline clinical characteristics between patients with and without ISR, other than patient age.

**Table 1:** Baseline clinical characteristics and medications at the time of follow-up between patients with and without in-stent restenosis.

	Total population	ISR (+) (n = 14)	ISR (-) (n = 112)	p value
**Demographic findings**				
Age (years)	60.5±10.0	65.6±11.2	59.8±9.7	0.041
Sex (males, %)	86.5	85.7	86.6	0.927
Clinical diagnosis (%)				0.966
Stable angina	63.5	64.3	63.4	
Unstable angina	27.0	28.6	28.6	
NSTEMI	7.9	7.1	6.3	
STEMI	1.6	0.0	1.8	
Hypertension (%)	63.5	71.4	62.5	0.513
Diabetes (%)	35.7	42.9	34.8	0.554
Smoking (%)				0.344
Current smoker	46.0	42.9	46.6	
Ex-smoker	32.5	21.4	33.9	
Never smoker	21.4	35.7	19.6	
Dyslipidemia (%)	42.9	42.9	42.9	1.000
Previous MI (%)	10.3	0.0	11.6	0.178
**Laboratory findings**				
Total cholesterol (mg/dL)	182±38	180±42	183±38	0.625
Triglyceride (mg/dL)	155±86	155±80	155±87	0.840
HDL-cholesterol (mg/dL)	43±12	42±13	43±12	0.831
LDL-cholesterol (mg/dL)	103±30	97±29	104±31	0.400
Serum creatinine (mg/dL)	1.07±0.32	1.15±0.28	1.07±0.32	0.347
hsCRP (mg/dL)	1.02±2.14	0.56±0.75	1.10±2.27	0.633
**Functional tests**				
LV ejection fraction (%)	57.2±11.2	61.6±7.7	56.7±11.5	0.139
RWMA	35 (29.4%)	3 (25.0%)	32 (29.9%)	0.724
Q-wave in ECG	30 (24.0%)	4 (28.6%)	26 (23.4%)	0.671
**Medication at follow-up**				
Aspirin	126 (100%)	14 (100%)	112 (100%)	NA
Clopidogrel	123 (97.6%)	13 (92.9%)	110 (98.2%)	0.215
ACE inhibitor or ARB	90 (71.4%)	12 (85.7%)	78 (69.6%)	0.209
Statin	113 (89.7%)	14 (100%)	99 (88.4%)	0.178

ACE, angiotensin-converting enzyme; ARB, angiotensin-receptor blocker; ECG, electrocardiography; HDL, high density lipoprotein; hsCRP, high-sensitivity C-reactive protein; LDL, low density lipoprotein; LV, left ventricular; MI, myocardial infarction; NSTEMI, non-ST-segment elevation myocardial infarction; RWMA, regional wall motion abnormality; STEMI, ST-segment elevation myocardial infarction

### Angiographic and IVUS findings

On average, 1.7 ± 0.7 stents, with a total length of 46.4 ± 20.3 mm, were used to treat each lesion. Angiographic characteristics showed that the diameter of the most distally inserted stent was smaller in the ISR group than in those not exhibiting ISR; other characteristics were not significantly different between the groups (S6 Table). QCA revealed that patients with ISR had smaller post-PCI reference diameters and smaller MLDs than patients without ISR; however, diameter stenosis was similar between the groups (Table 2). At follow-up angiography, the ISR (+) patients had smaller reference diameters, MLDs, and diameter stenosis than did ISR (-) patients.

**Table 2:** Angiographic and quatitative coronary angiography characteristics between patients with and without in-stent restenosis.

	ISR (+) (n = 14)	ISR (-) (n = 112)	P value
**Angiographic characteristics**			
Total number of stents inserted	2.0±0.8	1.7±0.7	0.148
Total stent length	55.6±26.0	45.2±19.4	0.070
Max. balloon expansion pressure	13.2±4.0	14.7±3.9	0.182
**QCA Characteristics**			
Pre-PCI reference diameter (mm)	2.76±0.43	2.89±0.51	0.341
Post-PCI reference diameter (mm)	2.57±0.30	2.83±0.42	0.022
Post-PCI MLD (mm)	2.24±0.27	2.53±0.40	0.009
Post-PCI diameter stenosis (%)	12.27±10.15	10.44±7.21	0.523
F/U reference diameter (mm)	2.52±0.34	2.76±0.44	0.044
F/U MLD (mm)	0.61±0.58	2.24±0.45	<0.001
F/U diameter stenosis (%)	75.2±23.0	18.5±12.1	<0.001

F/U, follow-up; ISR, in-stent restenosis; MLD, minimal luminal diameter; PCI, percutaneous coronary intervention; QCA, quantitative coronary angiography

A qualitative analysis of post-PCI IVUS showed no differences in incomplete apposition, tissue prolapse, or edge dissection between patients with or without ISR (Table 3). The ISR (+) group had a significantly smaller mean MSA (3.51 ± 1.01 mm vs. 4.40 ± 1.47 mm, p = 0.016) and SER (66.7 ± 29.3% vs. 75.8 ± 19.7%, p = 0.009) than did the ISR (-) group.

**Table 3:** Post-percutaneous coronary intervention intravascular ultrasonography characteristics between patients with and without in-stent restenosis.

	ISR (+) (n = 14)	ISR (-) (n = 112)	P value
Incomplete apposition	1 (7.1%)	4 (3.7%)	0.465
Proximal edge	1	3	
Stent body	0	1	
Distal edge	0	0	
Tissue prolapse	1 (7.1%)	14 (12.5%)	0.560
Edge dissection	0 (0.0%)	1 (0.9%)	1.000
Proximal edge	0	0	
Distal edge	0	1	
MSA (mm^2^)	3.51±1.01	4.40±1.47	0.016
EEMA at MSA site (mm^2^)	9.18±3.10	10.98±4.60	0.233
MSA/EEMA	41.4±13.4%	43.6±15.7%	0.786
SER	66.7±29.3%	75.8±19.7%	0.009
Nominal CSA of stent (mm^2^)	5.55±1.37	5.84±1.59	0.506
Plaque CSA behind stent (mm^2^)	5.67±2.68	6.57±3.81	0.393
Percentage area stenosis (%)	58.6±13.4	56.4±15.7	0.612

CSA, cross-sectional area; EEMA, external elastic membrane area; ISR, in-stent restenosis; MSA, minimal stent area; SER, stent expansion ratio

### Independent predictors of ISR in revascularized CTO lesions

**Fig 2:**
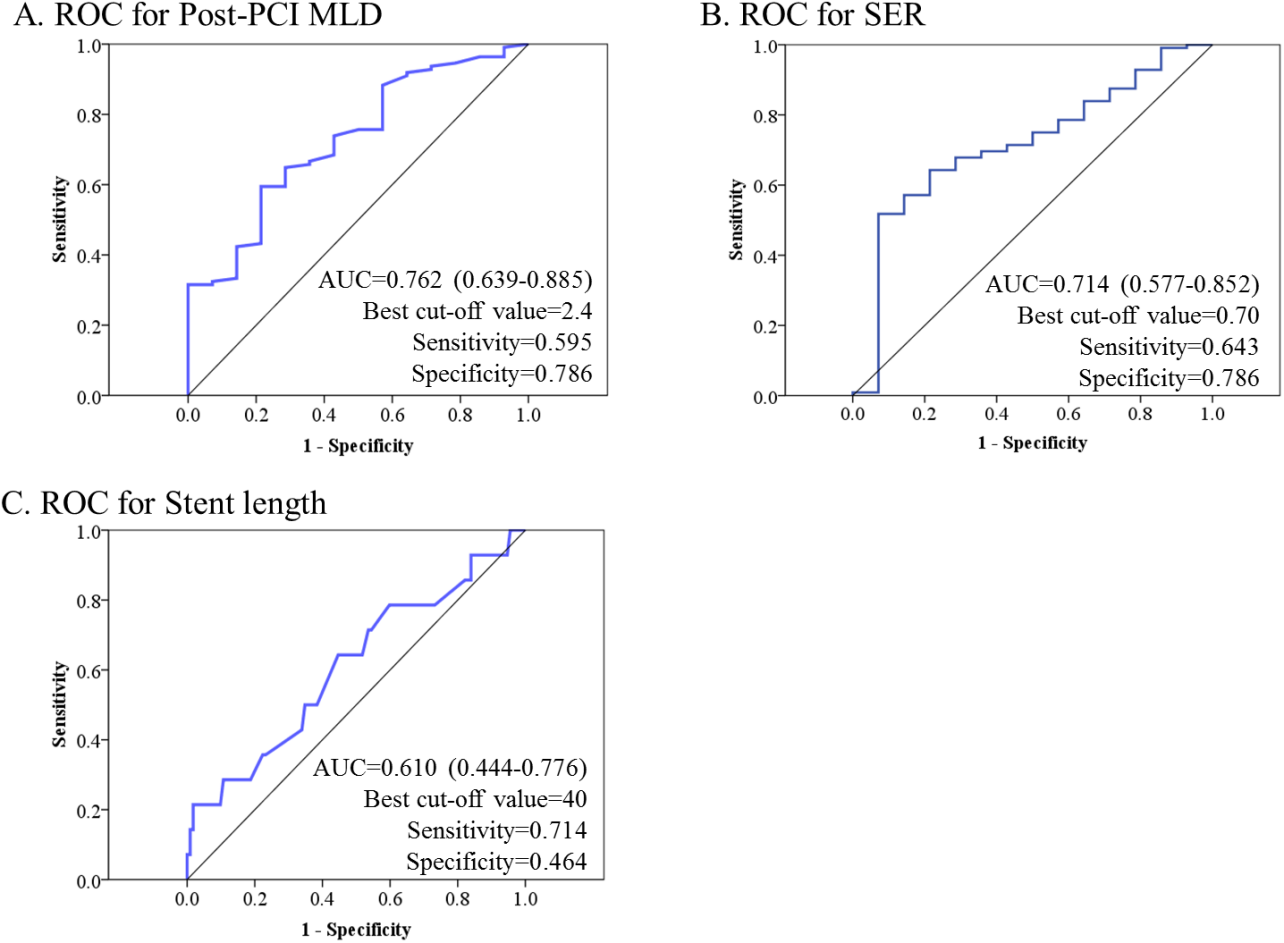
Optimal cut-off values. Optimal cut-off values were obtained using a receiver operating characteristics analysis for (A) post- percutaneous coronary intervention (PCI) minimal luminal diameter (MLD), (B) stent expansion ratio (SER), and (C) total stent length.

To investigate the factors associated with ISR, we included age, post-PCI MLD, and SER, along with the previously studied clinical risk factor (diabetes) and lesion factors (total stent length and severe calcification), in a multivariate analysis. These variables included those that were significant (p < 0.10) in the univariate analyses (i.e., age, distal stent diameter, post-PCI reference diameter, post-PCI MLD, MSA, and SER). Among these factors, MSA was correlated with SER (r = 0.653, p < 0.001). Additionally, the post-PCI reference diameter and distal stent diameter were correlated with post-PCI MLD (r = 0.866, p < 0.001 and r = 0.553, p < 0.001, respectively). To select the specific factors for multivariate analysis among those showing correlation, we chose those with large standard coefficients in the logistic regression model. Therefore, we used SER (standard coefficient, 0.126 vs. 0.087 for SER and MSA, respectively), and post-PCI MLD (standard coefficient, 0.085 vs. 0.079 vs. 0.020 for post-PCI MLD, post-PCI reference diameter, and distal stent diameter, respectively) rather than other correlated factors. We confirmed the adequacy of the model variables (e.g., dichotomous dependent variables, observation independence, linear relationships between continuous independent variables and the logit transformations of the dependent variables, multi-collinearity; S7 Table) and model calibration (Cox and Snell, R^2^ = 0.157; Nagelkerke, R^2^ = 0.312; Hosmer-Lemeshow, p = 0.645). Additionally, for post-PCI MLD, SER, and total stent length, we used the optimal cut-off values, which were 2.4 mm, 70%, and 40 mm, respectively (Fig 2). Multivariate analysis showed that a small post-PCI MLD (≤2.4 mm) and a low SER (≤70%) were factors significantly associated with ISR, whereas the clinical and lesion factors were insignificant (Table 4).

**Table 4:** Predictors of in-stent restenosis using logistic regression analysis.

Variables	Hazard ratio	95% Confidence Interval	P value	Standardized Coefficient
Age >60 years	0.712	0.189–2.687	0.617	0.017
Diabetes	1.087	0.304–3.888	0.898	-0.004
Total Stent length >40 mm	2.659	0.683–10.347	0.158	0.048
Severe calcification	1.882	0.259–13.672	0.532	0.016
Post-PCI MLD ≤2.4 mm	6.212	1.445–26.702	0.014	0.091
SER ≤70%	6.304	1.546–25.696	0.010	0.091

MLD, minimal luminal diameter; PCI, percutaneous coronary intervention; SER, stent expansion ratio

### Cut-off values of post-PCI MLD

**Fig 3:**
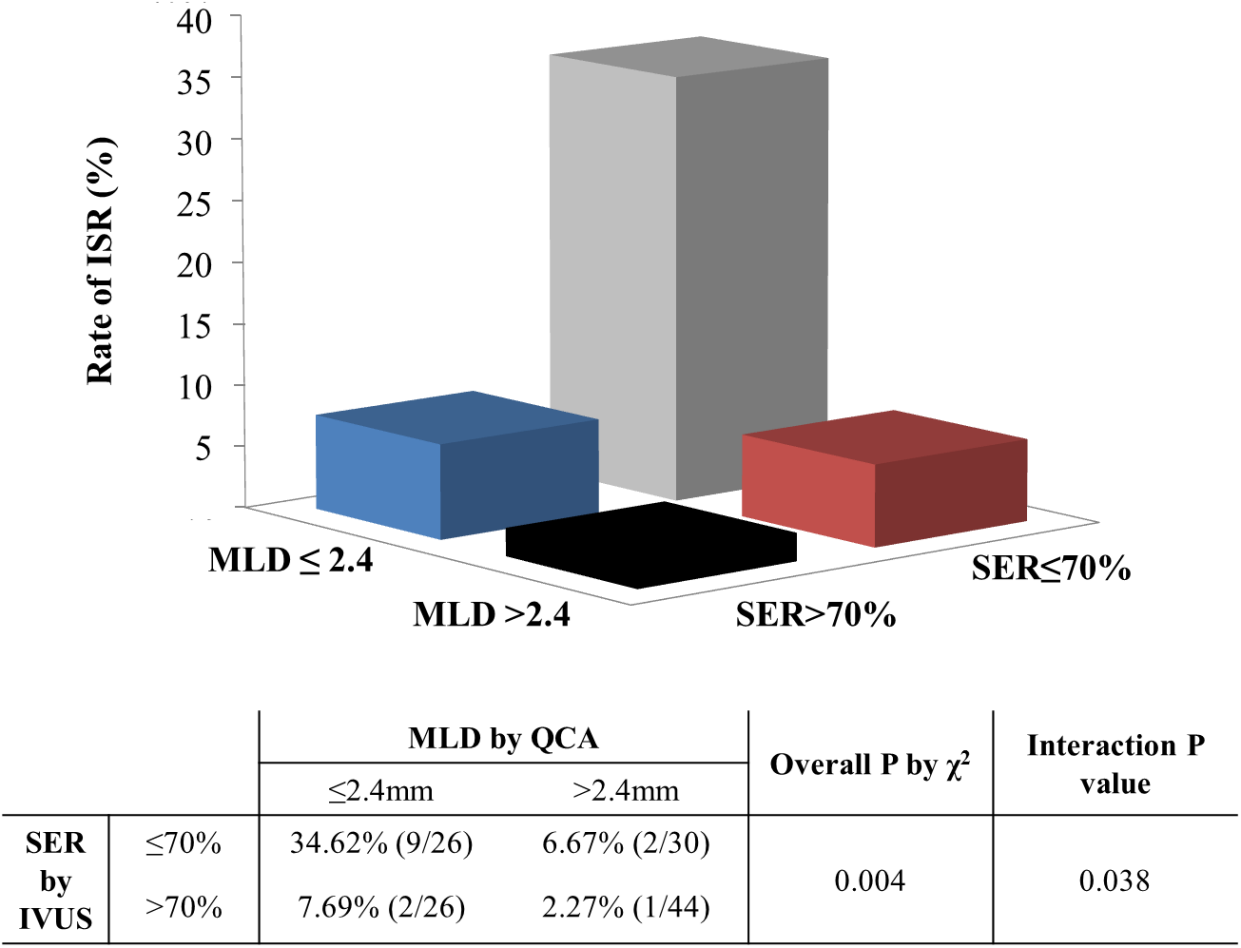
Frequency of in-stent restenosis (ISR), according to post-percutaneous coronary intervention (PCI) minimal luminal diameter (MLD) and stent expansion ratio (SER). Lesions with smaller post-PCI MLDs and SERs had significantly higher risks of ISR. Lesions with post-PCI MLDs ≤2.4 mm and SERs ≤70% were at the highest risk of ISR, with an odds ratio of 23.3 (95% confidence interval, 2.74–198.08) compared with lesions with larger MLDs and SERs. There was a significant interaction between post-PCI MLD and SER (p = 0.038) on ISR.

CTO lesions with post-PCI MLDs ≤2.4 mm and SERs ≤70% demonstrated significantly greater risk of ISR (19.6% [11/56] vs. 4.3% [3/70], p = 0.006, for post-PCI MLD ≤2.4 mm and >2.4 mm respectively; 21.2% [11/52] vs. 4.1% [3/74], p = 0.003, for SER ≤70% and >70%, respectively). Also, for the post-PCI MLD, the positive predictive value (PPV) was 19.6% (95% confidence interval [CI], 10.7–31.3%) and the negative predictive value (NPV) was 95.7% (95% CI, 89.3–98.9%). For the SER, the PPV was 21.2% (95% CI, 11.6–33.5%) and the NPV was 95.9% (95% CI, 89.8–99.0%). After combining the two criteria, the PPV was 34.6% (95% CI, 18.4–53.7%) and the NPV was 95.0% (95% CI, 89.6–98.2%). There was a significant interaction between the post-PCI MLD and SER (p = 0.038) for ISR. Furthermore, lesions with post-PCI MLDs ≤2.4 mm and SERs ≤70% were at the highest risk of ISR, with an odds ratio of 23.3 (95% CI, 2.74–198.08), compared with lesions having larger MLDs and SERs (Fig 3).

Among the 14 ISR (+) patients, 9 had post-PCI MLDs ≤2.4 mm and SERs ≤70%. Compared to these patients, those with post-PCI MLDs >2.4 mm and SERs >70% were older, had lower ejection factions, and their lesions tended to have smaller MSAs and lower maximum applied stent inflation pressures (S8 Table).

### ISR among non-AMI patients

In our study population, the CTO lesions were in non-target vessels, and were typically treated in staged procedures in patients with AMI. After excluding 10 AMI patients, the non-AMI group included 116 evaluable patients. For these patients, the baseline characteristics and QCA and IVUS analysis results are shown in S9 and S10 Tables. The study population, excluding those with AMIs, had similar baseline, QCA, and IVUS characteristics compared with those of the total population. We performed a multiple logistic regression analysis to identify those variables influencing the ISR risk exclusively in non-AMI patients. There was no multi-collinearity between the factors included in the analysis; and the model calibration was checked using the Cox and Snell R-Square (0.183), Nagelkerke R-square (0.363), and Hosmer-Lemeshow goodness-of-fit (p = 0.572) tests. As shown in Table 5, the total stent length, post-PCI MLD, and SER values were predictors of ISR, whereas the post-PCI MLD and SER were the two most powerful predictors, according to the standardized coefficients.

**Table 5:** Predictors of in-stent restenosis in non-acute myocardial infarction patients.

Variables	Hazard ratio	95% Confidence Interval	P value	Standardized coefficient
Age >60 years	1.736	0.396–7.602	0.464	0.028
Diabetes	1.336	0.321–5.559	0.691	0.014
Total Stent length >40 mm	5.046	1.042–24.445	0.044	0.081
Calcification	1.611	0.212–12.245	0.645	0.013
Post-PCI MLD ≤2.4 mm	7.449	1.534–36.172	0.013	0.101
SER ≤70%	12.273	2.254–66.816	0.004	0.127

MLD, minimal luminal diameter; PCI, percutaneous coronary intervention; SER, stent expansion ratio

## Discussion

To our knowledge, this is the first study involving the use of post-PCI IVUS to determine the predictors of ISR in patients with CTO lesions (11% of the current study’s population). Investigating clinical, lesion-related, and procedural characteristics, only procedural factors (post-PCI MLD ≤2.4 mm and SER ≤70%) were found to be predictors of ISR. CTO lesions exhibiting both post-PCI MLDs ≤2.4 mm and SERs ≤70% had a 15.3-fold higher risk of ISR, compared with lesions with larger post-PCI MLDs and SERs.

### Characteristics of CTO lesions as PCI targets

A coronary CTO may be defined as a long lesion having a heavy arterial atherosclerotic plaque burden that results in complete vessel occlusion lasting ≥3 months [15]. CTO lesions are unique in their lesion-related (plaque composition, complex morphology), procedure-related (different revascularization techniques), and patient-related (comorbidities) characteristics. Additionally, their associated angioplasty risks of coronary perforation, contrast nephropathy, radiation exposure, and collateral loss are unique [2]. Although collaterals may develop to distal vessels, insufficient blood flow to the myocardial bed results in ischemia and angina symptoms [11]. CTO is quite common, with an incidence as high as 15–30%, and these lesions have been found in approximately 50% of patients with significant coronary artery disease [14]. By adapting and refining advanced PCI techniques, PCI for CTO lesions has become the “last frontier” of interventional cardiology. However, CTO lesions have a comparatively high risk of ISR, compared with non-occlusive stenosis [3]. Although drug-eluting stents have significantly reduced restenosis, CTO lesions continue to exhibit higher ISR rates than those associated with new generation stents in patients with non-CTO lesions [16]. Moreover, ISR is a significant limitation of contemporary PCI that may present as unstable angina (16–66%) or myocardial infarction (1–20%) [4]. Therefore, in this study, we investigated the predictors of ISR in patients undergoing PCI for CTO lesions.

### Predictors of ISR after CTO recanalization

The IVUS-measured predictors of ISR after PCI in non-CTO lesions, such as a small MSA [17] and long stent length [3], have been studied. However, few studies have evaluated post-PCI outcome predictors exclusively for CTO lesions, and these studies have been limited to clinical or lesion-related characteristics (i.e., complex lesion morphology or comorbidities, such as diabetes mellitus) [18, 19]. Noguchi et al. reported calcification and CTO length as ISR predictors [20], whereas Valenti et al. reported that specific techniques (the subintimal tracking and re-entry technique) and specific stents (everolimus-eluting stents) were associated with ISR [5]. Additionally, using QCA, post-procedural MLDs and stent lengths have been reported to be factors predicting ISR [16]. Thus, various clinical, lesion-related, and procedural factors have been claimed to be ISR predictors, but without any clear consistency across studies.

We performed IVUS analyses to obtain precise information regarding the unique characteristics of CTO lesions, and examined previously reported clinical, lesion-related, and procedural factors suggested to predict ISR. In our study population, patients demonstrating ISR were older than those without ISR. Previous reports also indicated that patients with ISR are older than those without ISR, but advanced age was not found to be a significant predictor of ISR. Kastrati et al., in a study to identify factors predictive of restenosis, reported that patients demonstrating ISR were older (67.1 ± 10.3 years) than those without ISR (65.3 ± 10.2 years, p = 0.02), whereas old age was not an independent predictor of ISR (p = 0.11) [21]. Although not exhibiting a significant difference, Hong et al. also reported that patients with ISR were older (59 ± 8 years) than those without ISR (58 ± 10 years) [3]. Patients with ISR also had longer stent lengths, smaller post-PCI reference diameters and MLDs, and smaller MSA and SER values. However, after multivariate analysis, no clinical or lesion-related characteristics were found to be predictive of ISR, but procedural characteristics, such as post-PCI MLD and SER, were predictive.

In the current study, post-PCI MLD was measured using QCA and was a pre-evaluated factor, consistent with previous reports [16]; it is also a factor known to be associated with ISR in non-CTO lesions [22]. SER is a reformulated variable measured using IVUS, which is somewhat related to the MSA, and is a well-known factor for ISR in non-CTO lesions [17]. Thus, both a large post-PCI MLD and a large expansion ratio should be achieved for efficacious CTO treatment. Further, post-PCI MLD, SER, and the combination of the two factors showed low PPVs and high NPVs, possibly resulting from the low prevalence of ISR. In clinical practice, patients with large post-PCI MLDs and large SERs may have a minimal risk of ISR.

However, 5 ISR(+) patients had a post-PCI MLD >2.4 mm or a SER >70%. That is, ISR occurred despite these patients receiving procedures that satisfied the standards of our study. Therefore, sufficient revascularization does not guarantee an ISR-free state. Additional procedures, such as adjunctive balloon dilatation, could be performed to reduce ISR, but achieving these SER and MLD standards, in some CTO lesions, might not be possible because of heavy plaque burdens and vessel rigidity.

Moreover, our results provide further insights into the impact of procedural characteristics. Although clinical factors, such as age and diabetes status, and lesion-related factors, such as calcification and lesion length, are known to predict restenosis, our results show that procedural characteristics are predictive. This may be the result of various causes, including well-treated clinical risk factors, superior performance of drug eluting stents, and the potential importance of favorable procedural results. Taken together, our results further stress the need for meticulous technique when performing percutaneous coronary interventions in patients with CTO lesions. A previous report assessed the predictors of target lesion revascularization from a prospective, multicenter study conducted at 41 American centers and involving 1557 consecutive patients. That report revealed that less acute gain was the exclusive predictor of target lesion revascularization, whereas other factors, such as diabetes mellitus, stent length, smoking, etc., were not predictive [23]. Consistent with our results, impact of procedural factors was stressed over clinical factors.

### Standard for optimal expansion

SER is a calculated variable that reflects the optimal expansion ratio. Although the importance of stent optimization has been previously emphasized [24, 25], a consensus definition of “adequate” expansion is still lacking. In the MUSIC criteria, “adequate” expansion was defined as >90% of the average reference CSA, and in the HOME DES trial, optimal stent deployment was defined as complete apposition of the stent struts, the absence of edge dissection, and adequate stent expansion, defined as either an MSA >5.0 mm^2^ or >90% of the distal reference lumen area [26]. Additionally, the AVIO study defined “adequate expansion” as an MSA >70% of the chosen balloon [27]. In our study, using receiver operating characteristic analysis, an expansion of 70% of the nominal stent CSA was the best value for predicting a decreased risk of ISR, similar to the AVIO study. According to our results, adequate expansion of CTO lesions can significantly reduce the risk of ISR.

### Analysis in non-AMI patients

Unlike the total population where post-PCI MLDs and SERs were predictors of ISR, the total stent length was a significant predictor of ISR in non-AMI patients. Previous reports have also shown that stent length is a well-known predictor of ISR, both in standard stenotic coronary lesions and CTO lesions [4, 16]. Although not significant in the total population, total stent length was the third strongest ISR predictor, according to the standardized coefficient. The distinct nature of AMI patients may have diluted the effect of stent length; however, the small number of AMI patients involved in this study does not allow a definite conclusion to be drawn regarding the differential effects of stent length on ISR, according to clinical presentation. Additional large-scale studies investigating the predictors of ISR need to be conducted exclusively in non-AMI patients.

## Limitations

Our study has several limitations. First, there may have been a selection bias regarding patients receiving follow-up coronary angiography. Although there were no significant differences between the groups, except for the patients not receiving follow-up coronary angiography being older, we cannot exclude the potential of a hidden selection bias. Second, a limited number of study events were analyzed due to the small population size and the short follow-up duration. This is a potential problem that might have influenced the adequacy of the multivariate logistic analysis model (over-fitting of the statistical model) and the generalization of this research. An independent study may be necessary to verify our findings prior to the clinical application of this study to current practice. Moreover, 96% of the present population underwent PCI via an antegrade approach, suggesting some degree of selection and perhaps lower case complexity, thereby biasing the results. Third, most CTO cases in this study involved the left anterior descending artery, unlike recent reports showing that the right coronary artery is the most common vessel exhibiting CTO [14]. This may be explained by local population variations in Northeast Asia [7, 28] or by the small sample size of our study. Lastly, ISR was defined as >50% diameter stenosis observed using angiography, whereas there is growing evidence that morphology is inadequate to evaluate stenosis. A recent study assessed the clinical and morphological predictors for functionally significant ISR, defined as lesions that were single-photon emission computed tomography-positive or had fractional flow reserves <0.75–0.80 [29]. Further studies may be necessary for predicting functional ISR after revascularization of CTO lesions.

## Conclusion

In coronary angiographs taken 9 months after PCI of CTO lesions, ISR occurred in 11% of patients. A post-PCI MLD ≤2.4 mm and a SER ≤70% were both independent predictors of ISR. Thus, not only a large post-PCI MLD, but also a large expansion ratio should be achieved to increase the chance of an efficacious CTO treatment.

## Supporting Information

S1 Fig (A) Bland–Altman plot for inter-observer variability of intravascular ultrasonography variables; (B) Bland–Altman plot for inter-observer variability of quantitative coronary angiography variables. The Bland–Altman plot indicated excellent agreement between the two observers. (TIF)

S2 Fig Bland–Altman plot for intra-observer variability. A Bland–Altman plot showed excellent agreement between two measurements for one observer.
(TIF)

S1 Table Baseline clinical characteristics of patients undergoing and not undergoing follow-up coronary angiography. (DOCX)

S2 Table Mean difference between two measurements for inter-observer variability. (DOCX)

S3 Table Intraclass correlation coefficient for inter-observer variability. (DOCX)

S4 Table Mean difference between two measurements for intra-observer variability (DOCX)

S5 Table Intraclass correlation coefficient for intra-observer variability. (DOCX)

S6 Table Angiographic and stent-related characteristics between patients with and without in-stent restenosis. (DOCX)

S7 Table Multicollinearity test results. (DOCX)

S8 Table Comparison of patients with quantitative coronary angiography ‘post-percutaneous coronary intervention (PCI) minimal luminal diameters (MLDs) ≤2.4 mm and stent expansion ratios (SERs) ≤70%’ and ‘post-PCI MLDs >2.4 mm and/or SERs >70%.’ (DOCX)

S9 Table Baseline clinical characteristics and medication at follow-up, between patients with and without in-stent restenosis, in non-AMI patients. (DOCX)

S10 Table Angiographic, QCA and Post-PCI IVUS characteristics between patients with and without in-stent restenosis, in non-AMI patients. (DOCX)
