# Supplementary figures and images for: Intravascular Ultrasound and Angiographic Predictors of In-Stent Restenosis of Chronic Total Occlusion Lesions
Source: PLoS One. 2015 Oct 14;10(10):e0140421. doi: 10.1371/journal.pone.0140421 (PMC4605613; doi:10.1371/journal.pone.0140421)

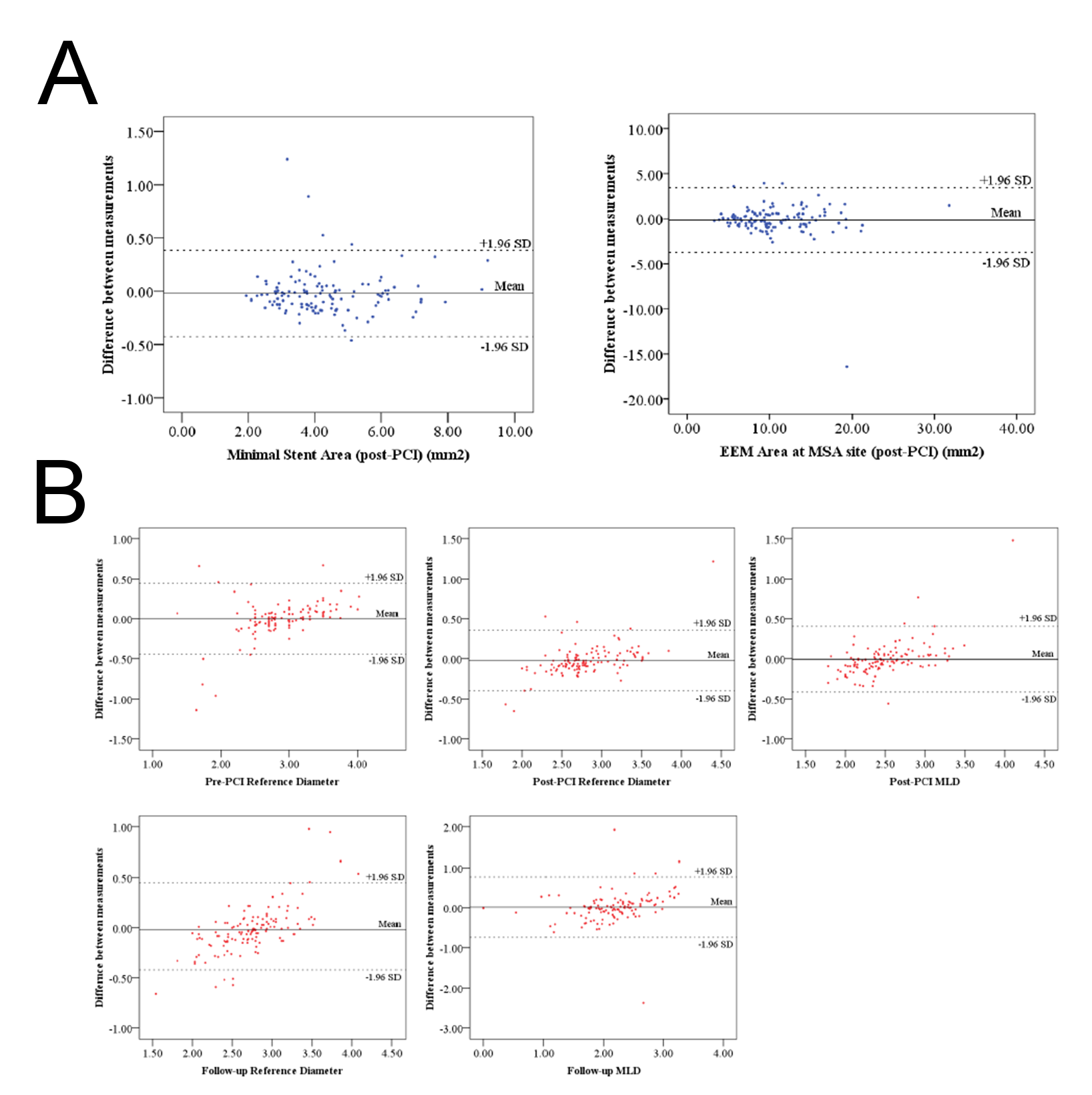

Supplement: S1 Fig — (TIF) [file pone.0140421.s001.tif]

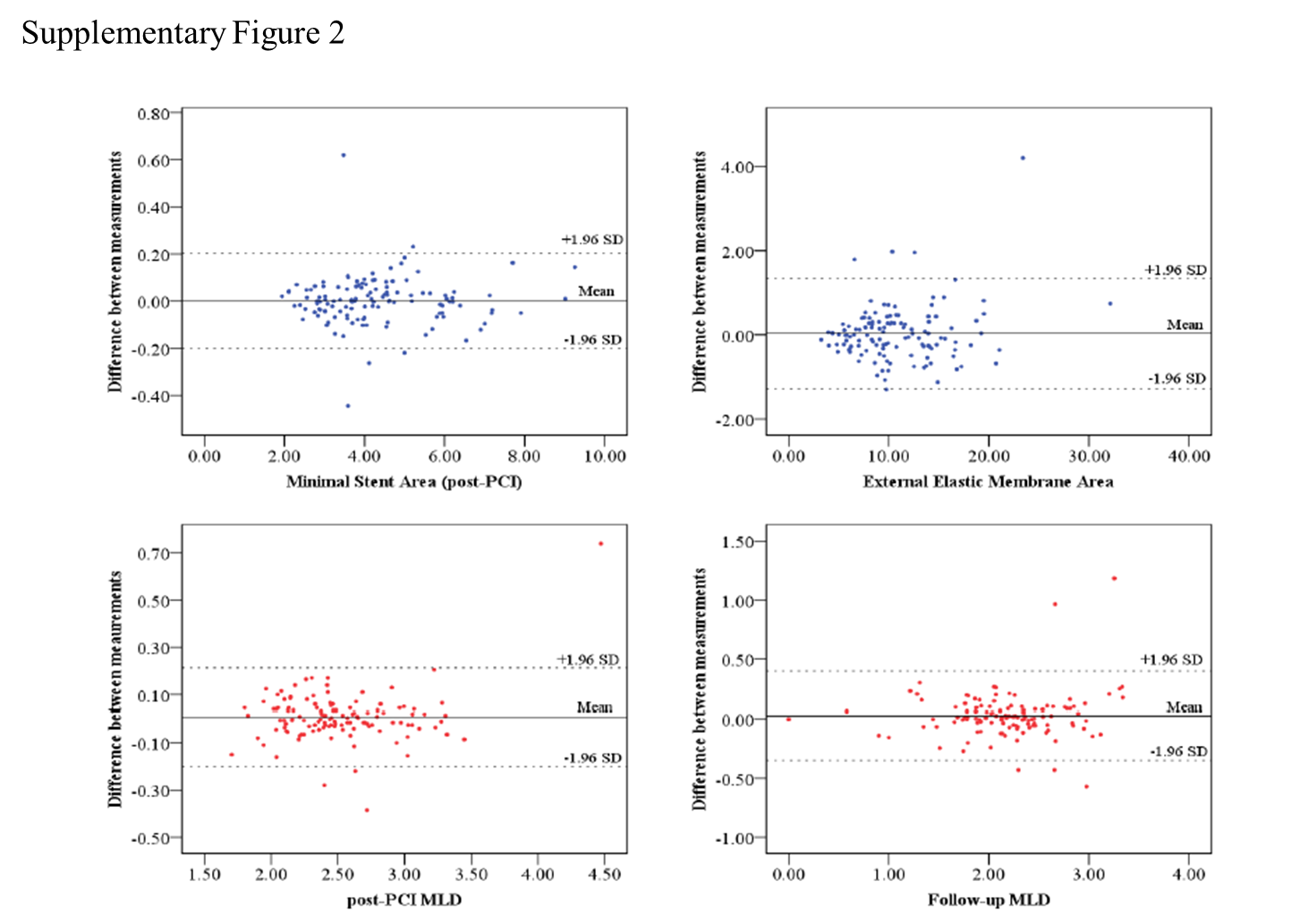

Supplement: S2 Fig — A Bland–Altman plot showed excellent agreement between two measurements for one observer. (TIF) [file pone.0140421.s002.tif]
