# Supplementary material for: Intravascular Ultrasound and Angiographic Predictors of In-Stent Restenosis of Chronic Total Occlusion Lesions
Source: PLoS One. 2015 Oct 14;10(10):e0140421. doi: 10.1371/journal.pone.0140421 (PMC4605613; doi:10.1371/journal.pone.0140421)
Supplement: S1 Table — (DOCX) [file pone.0140421.s003.docx]

**S1 Table. Baseline clinical characteristics of patients undergoing and not undergoing follow-up coronary angiography.**

|  | Follow-up CAG (+)  (n=126) | Follow-up CAG (-)  (n=43) | P |
| --- | --- | --- | --- |
| **Age (years old)** | 60.5±10.0 | 66.7±11.0 | 0.001 |
| **Sex (Male ratio, %)** | 86.5 | 76.7 | 0.131 |
| **Clinical diagnosis (%)** |  |  | 0.719 |
| **Stable Angina** | 63.5 | 60.5 |  |
| **Unstable Angina** | 28.6 | 27.9 |  |
| **NSTEMI** | 6.3 | 7.0 |  |
| **STEMI** | 1.6 | 4.7 |  |
| **Hypertension (%)** | 63.5 | 65.1 | 0.848 |
| **Diabetes (%)** | 35.7 | 37.2 | 0.860 |
| **Smoking (%)** |  |  | 0.541 |
| **Current smoker** | 46.0 | 37.2 |  |
| **Ex-smoker** | 32.5 | 58.1 |  |
| **Never smoker** | 21.4 | 4.7 |  |
| **Dyslipidemia (%)** | 42.9 | 46.5 | 0.677 |
| **Previous MI (%)** | 10.3 | 16.3 | 0.296 |
| **Total cholesterol (mg/dl)** | 182±38 | 176±42 | 0.343 |
| **Triglyceride (mg/dl)** | 155±86 | 147±155 | 0.686 |
| **HDL-cholesterol (mg/dl)** | 43±12 | 45±12 | 0.305 |
| **LDL-cholesterol (mg/dl)** | 103±30 | 98±28 | 0.400 |
| **Serum Creatinine (mg/dl)** | 1.07±0.32 | 1.34±1.12 | 0.138 |
| **hsCRP (mg/dl)** | 1.02±2.14 | 0.88±1.26 | 0.694 |
| **LV ejection fraction (%)** | 57.2±11.2 | 54.9±10.0 | 0.244 |
| **Lesion location (%)** |  |  | 0.061 |
| **LAD** | 46.0 | 25.6 |  |
| **LCX** | 20.6 | 32.6 |  |
| **RCA** | 33.3 | 41.9 |  |

CAG, coronary angiography; NSTEMI, non-ST-segment elevation myocardial infarction; STEMI, ST-segment elevation myocardial infarction; MI, myocardial infarction; HDL, high density lipoprotein; LDL, low density lipoprotein; hsCRP, high-sensitivity C-reactive protein; LV, left ventricular; LAD, left anterior descending artery; LCX, left circumflex artery; RCA, right coronary artery.
