# Supplementary material for: Intravascular Ultrasound and Angiographic Predictors of In-Stent Restenosis of Chronic Total Occlusion Lesions
Source: PLoS One. 2015 Oct 14;10(10):e0140421. doi: 10.1371/journal.pone.0140421 (PMC4605613; doi:10.1371/journal.pone.0140421)
Supplement: S2 Table — (DOCX) [file pone.0140421.s004.docx]

**S2 Table. Mean difference between two measurements for inter-observer variability.**

|  |  | Difference | P value |
| --- | --- | --- | --- |
| IVUS variables | Minimal Stent Area | -0.0199±0.206 | 0.280 |
|  | External Elastic Membrane Area | -0.146±1.83 | 0.371 |
| QCA variables | Pre-PCI Reference Diameter | 0.003±0.227 | 0.885 |
|  | Post-PCI Reference Diameter | -0.0198±0.193 | 0.252 |
|  | Post-PCI Minimal Luminal Diameter | -0.005±0.211 | 0.787 |
|  | Follow-up Reference Diameter | -0.0195±0.240 | 0.362 |
|  | Follow-up Minimal Luminal Diameter | 0.0181±0.384 | 0.598 |
