# Supplementary material for: Intravascular Ultrasound and Angiographic Predictors of In-Stent Restenosis of Chronic Total Occlusion Lesions
Source: PLoS One. 2015 Oct 14;10(10):e0140421. doi: 10.1371/journal.pone.0140421 (PMC4605613; doi:10.1371/journal.pone.0140421)
Supplement: S3 Table — (DOCX) [file pone.0140421.s005.docx]

**S3 Table. Intraclass correlation coefficient for inter-observer variability.**

|  |  | Intraclass Correlation Coefficient |
| --- | --- | --- |
| IVUS variables | Minimal Stent Area | 0.995 (0.993-0.996) |
|  | External Elastic Membrane Area | 0.958 (0.941-0.971) |
| QCA variables | Pre-PCI Reference Diameter | 0.949 (0.927-0.964) |
|  | Post-PCI Reference Diameter | 0.946 (0.923-0.962) |
|  | Post-PCI Minimal Luminal Diameter | 0.931 (0.901-0.951) |
|  | Follow-up Reference Diameter | 0.923 (0.891-0.946) |
|  | Follow-up Minimal Luminal Diameter | 0.921 (0.887-0.944) |
