# Supplementary material for: Intravascular Ultrasound and Angiographic Predictors of In-Stent Restenosis of Chronic Total Occlusion Lesions
Source: PLoS One. 2015 Oct 14;10(10):e0140421. doi: 10.1371/journal.pone.0140421 (PMC4605613; doi:10.1371/journal.pone.0140421)
Supplement: S4 Table — (DOCX) [file pone.0140421.s006.docx]

**S4 Table. Mean difference between two measurements for intra-observer variability**

|  |  | Difference | P value |
| --- | --- | --- | --- |
| IVUS variables | Minimal Stent Area | 0.0017±0.103 | 0.850 |
|  | External Elastic Membrane Area | 0.0275±0.666 | 0.644 |
| QCA variables | Post-PCI Minimal Luminal Diameter | 0.0074±0.105 | 0.431 |
|  | Follow-up Minimal Luminal Diameter | 0.0260±0.191 | 0.129 |
