# Supplementary material for: Intravascular Ultrasound and Angiographic Predictors of In-Stent Restenosis of Chronic Total Occlusion Lesions
Source: PLoS One. 2015 Oct 14;10(10):e0140421. doi: 10.1371/journal.pone.0140421 (PMC4605613; doi:10.1371/journal.pone.0140421)
Supplement: S5 Table — (DOCX) [file pone.0140421.s007.docx]

**S5 Table. Intraclass correlation coefficient for intra-observer variability.**

|  |  | Intraclass Correlation Coefficient |
| --- | --- | --- |
| IVUS variables | Minimal Stent Area | 0.999 (0.998-0.999) |
|  | External Elastic Membrane Area | 0.990 (0.986-0.993) |
| QCA variables | Post-PCI Minimal Luminal Diameter | 0.983 (0.976-0.988) |
|  | Follow-up Minimal Luminal Diameter | 0.981 (0.973-0.986) |
