# Supplementary material for: Intravascular Ultrasound and Angiographic Predictors of In-Stent Restenosis of Chronic Total Occlusion Lesions
Source: PLoS One. 2015 Oct 14;10(10):e0140421. doi: 10.1371/journal.pone.0140421 (PMC4605613; doi:10.1371/journal.pone.0140421)
Supplement: S6 Table — (DOCX) [file pone.0140421.s008.docx]

**S6 Table. Angiographic and stent-related characteristics between patients with and without in-stent restenosis.**

| Restenosis | ISR (+) (n=14) | ISR (-) (n=112) | P value |
| --- | --- | --- | --- |
| **Angiographic findings** |  |  |  |
| Lesion location (%) |  |  | 0.371 |
| LAD | 35.7 | 47.3 |  |
| LCX | 14.3 | 21.4 |  |
| RCA | 50.0 | 31.3 |  |
| Disease extent (1VD/2VD/3VD, %) | 21.4 / 35.7 / 42.9 | 25.0 / 42.0 / 33.0 | 0.766 |
| **CTO morphology** |  |  |  |
| Blunt stump | 3 (21.4%) | 37 (33.0%) | 0.379 |
| Bridging collateral | 1 (7.1%) | 9 (8.0%) | 0.907 |
| Side branch | 8 (57.1%) | 77 (68.8%) | 0.382 |
| Size < 1.5mm | 5 (35.7%) | 53 (47.3%) |  |
| Size ≥ 1.5mm | 2 (14.3%) | 20 (17.9%) |  |
| Mixed | 1 (7.1%) | 4 (3.6%) |  |
| Trifurcation | 2 (14.3%) | 8 (7.1%) | 0.351 |
| Severe tortuosity | 3 (21.4%) | 18 (16.1%) | 0.612 |
| Calcification* | 85.7 / 0.0 / 14.3 | 83.9 / 9.8 / 6.3 | 0.286 |
| Thrombus | 2 (14.3%) | 7 (6.3%) | 0.271 |
| Collateral grade† | 35.7 / 64.3 / 0.0 | 45.5 / 50.9 / 3.6 | 0.550 |
| **Stent characteristics** |  |  |  |
| Stent generation |  |  | 0.824 |
| 1^st^ generation DES | 9 (64.3%) | 71 (63.4%) |  |
| 2^nd^ generation DES | 5 (35.7%) | 38 (33.9%) |  |
| 3^rd^ generation DES | 0 (0.0%) | 3 (2.7%) |  |
| Stent number | 2.0±0.8 | 1.7±0.7 | 0.155 |
| Stent total length (mm) | 55.6±26.0 | 45.2±19.4 | 0.180 |
| Stent Length >40mm | 10 (71.4%) | 60 (53.6%) | 0.205 |
| Stent diameter |  |  |  |
| Most Proximal stent | 3.36±1.00 | 3.65±1.16 | 0.366 |
| Most Distal stent | 2.14±0.77 | 2.72±1.12 | 0.021 |
| Stent at MSA site | 2.50±1.02 | 2.73±1.14 | 0.469 |
| Maximum inflation pressure to Stent | 13.2±4.0 | 14.7±3.9 | 0.182 |

ISR, in-stent restenosis; LAD, left anterior descending coronary artery; LCX, left circumflex coronary artery; RCA, right coronary artery; VD, vessel disease; DES, drug eluting stent; MSA, minimal stent area; CSA, cross-sectional area

***** Calcification: None or mild / Moderate / Severe

**†** Collateral grade: Grade 1 / Grade 2 / Grade 3
