# Supplementary material for: Intravascular Ultrasound and Angiographic Predictors of In-Stent Restenosis of Chronic Total Occlusion Lesions
Source: PLoS One. 2015 Oct 14;10(10):e0140421. doi: 10.1371/journal.pone.0140421 (PMC4605613; doi:10.1371/journal.pone.0140421)
Supplement: S7 Table — (DOCX) [file pone.0140421.s009.docx]

**S7 Table. Multicollinearity test results.**

| Factor | VIF* |
| --- | --- |
| Age | 1.066 |
| Diabetes | 1.008 |
| Calcification | 1.100 |
| Stent total length | 1.099 |
| Minimal stent area | 1.295 |
| Stent expansion ratio | 1.393 |

* VIF: Variance inflation factor
