# Supplementary material for: Intravascular Ultrasound and Angiographic Predictors of In-Stent Restenosis of Chronic Total Occlusion Lesions
Source: PLoS One. 2015 Oct 14;10(10):e0140421. doi: 10.1371/journal.pone.0140421 (PMC4605613; doi:10.1371/journal.pone.0140421)
Supplement: S8 Table — (DOCX) [file pone.0140421.s010.docx]

**S8 Table. Comparison of patients with quantitative coronary angiography ‘post-percutaneous coronary intervention (PCI) minimal luminal diameters (MLDs) ≤2.4 mm and stent expansion ratios (SERs) ≤70%’ and ‘post-PCI MLDs >2.4 mm and/or SERs >70%.’**

|  | Post-PCI MLD≤2.4mm and SER≤70% (n=9) | Others (n=5) | P value |
| --- | --- | --- | --- |
| **Demographic findings** |  |  |  |
| Age (years old) | 61.8±9.4 | 72.4±11.9 | 0.089 |
| Sex (male ratio, %) | 77.8 (7/9) | 100 (5/5) | 0.255 |
| Hypertension (%) | 77.8 (7/9) | 60 (3/5) | 0.480 |
| Diabetes (%) | 55.6 (5/9) | 20 (1/5) | 0.198 |
| Smoking (%) | 44.4 / 22.2 / 33.3 | 40 / 20 / 40 | 0.969 |
| Dyslipidemia (%) | 44.4 (4/9) | 40 (2/5) | 0.872 |
| Previous MI (%) | 100 (9/9) | 100 (5/5) | NA |
| Clinical diagnosis (%) |  |  | 0.238 |
| Stable Angina | 77.8 (7/9) | 40 (2/5) |  |
| Unstable Angina | 22.2 (2/9) | 40 (2/5) |  |
| NSTEMI | 0 | 20 (1/5) |  |
| STEMI |  |  |  |
| **Laboratory findings** |  |  |  |
| Total cholesterol (mg/dl) | 183±51 | 173±18 | 0.684 |
| Triglyceride (mg/dl) | 154±47 | 158±128 | 0.940 |
| HDL-cholesterol (mg/dl) | 40±12 | 45±14 | 0.516 |
| LDL-cholesterol (mg/dl) | 101±33 | 91±20 | 0.549 |
| Serum Creatinine (mg/dl) | 1.08±0.31 | 1.27±0.19 | 0.246 |
| hsCRP (mg/dl) | 0.71±0.93 | 0.32±0.20 | 0.386 |
| **Functional tests** |  |  |  |
| LV ejection fraction (%) | **64.1±6.3** | **56.0±8.1** | **0.074** |
| RWMA (%) | 25 (2/8) | 25 (1/4) | 1.000 |
| Q wave in ECG (%) | 22.2 (2/9) | 40.0 (2/5) | 0.480 |
| **Angiographic findings** |  |  |  |
| Lesion location (%) |  |  | 0.218 |
| LAD | 44.4 (4/9) | 20.0 (1/5) |  |
| LCX | 22.2 (2/9) | 0.0 |  |
| RCA | 33.3 (3/9) | 80.0 (4/5) |  |
| Disease extent (1VD/2VD/3VD, %) | 22.2 / 33.3 / 44.4 | 20 / 40 / 40 | 0.969 |
| **CTO morphology** |  |  |  |
| Blunt stump (%) | 22.2 (2/9) | 20.0 (1/5) | 0.923 |
| Bridging collateral (%) | 0.0 (0/9) | 20.0 (1.5) | 0.164 |
| Side branch (%) | 44.4 (4/9) | 80 (4/5) | 0.198 |
| Size < 1.5mm | 22.2 (2/9) | 60.0 (3/5) |  |
| Size ≥ 1.5mm | 11.1 (1/9) | 20.0 (1/5) |  |
| Mixed | 11.1 (1/9) | 0.0 (0/5) |  |
| Trifurcation (%) | 11.1 (1/9) | 20 (1/5) | 0.649 |
| Severe tortuosity (%) | 22.2 (2/9) | 20 (1/5) | 0.923 |
| Calcification* (%) | 11.1 (1/9) | 20 (1/5) | 0.649 |
| Thrombus (%) | 22.2 (2/09) | 20 (1/5) | 0.255 |
| Collateral grade† (%) | 22.2 / 77.8 | 60 / 40 | 0.158 |
| **Stent characteristics** |  |  |  |
| Stent generation (%) |  |  | 0.803 |
| 1^st^ generation DES | 66.7 (6/9) | 60 (3/5) |  |
| 2^nd^ generation DES | 33.3 (3/9) | 40 (2/5) |  |
| Stent number | 1.9±0.8 | 2.2±0.8 | 0.499 |
| Stent total length (mm) | 53.3±24.8 | 59.8±30.5 | 0.673 |
| Stent Length >40mm (%) | 33.3 (3/9) | 20.0 (1/5) | 0.597 |
| Stent diameter |  |  |  |
| Proximal stent (mm) | 3.33±0.87 | 3.40±1.34 | 0.911 |
| Distal stent (mm) | 2.22±0.67 | 2.00±1.00 | 0.625 |
| Stent at ISR site (mm) | 2.78±0.97 | 2.00±1.00 | 0.181 |
| Nominal CSA of stent (mm^2^) | 5.89±1.49 | 4.95±0.98 | 0.232 |
| Maximun pressure to Stent (atm) | 11.9±4.1 | 15.6±2.6 | 0.094 |
| **QCA characteristics** |  |  |  |
| Pre-PCI Reference diameter (mm) | 2.80±0.36 | 2.68±0.57 | 0.633 |
| Post-PCI Reference diameter (mm) | 2.57±0.33 | 2.56±0.25 | 0.988 |
| Post-PCI MLD (mm) | 2.18±0.17 | 2.34±0.40 | 0.440 |
| Post-PCI Diameter stenosis (%) | 14.2±8.2 | 8.8±13.3 | 0.360 |
| F/U Reference diameter (mm) | 2.51±0.37 | 2.52±0.32 | 0.994 |
| F/U MLD (mm) | 0.91±1.01 | 0.66±0.72 | 0.635 |
| F/U Diameter stenosis (%) | 74.2±24.6 | 70.6±31.2 | 0.815 |
| **Post-PCI IVUS characteristics** |  |  |  |
| Incomplete Apposition |  |  | 0.164 |
| Proximal edge | 0% | 20.0% (1/5) |  |
| Stent body | 0% | 0% |  |
| Distal edge | 0% | 0% |  |
| Tissue prolapse | 11.1% (1/9) | 0% | 0.439 |
| Edge dissection | 0% | 0% |  |
| MSA (mm^2^) | 3.13±0.58 | 4.19±1.32 | 0.056 |
| EEMA at MSA site (mm^2^) | 9.16±2.93 | 9.22±3.75 | 0.973 |
| MSA/EEMA (%) | 37.4±12.8 | 48.5±12.4 | 0.142 |
| SER (%) | 54.5±10.2 | 88.7±40.6% | 0.134 |
| Plaque CSA behind stent (mm^2^) | 6.03±2.82 | 5.03±2.56 | 0.527 |
| Percent area stenosis (%) | 62.6±12.8 | 51.5±12.4 | 0.142 |
