# Supplementary material for: Intravascular Ultrasound and Angiographic Predictors of In-Stent Restenosis of Chronic Total Occlusion Lesions
Source: PLoS One. 2015 Oct 14;10(10):e0140421. doi: 10.1371/journal.pone.0140421 (PMC4605613; doi:10.1371/journal.pone.0140421)
Supplement: S9 Table — (DOCX) [file pone.0140421.s011.docx]

**S9 Table. Baseline clinical characteristics and medication at follow-up, between patients with and without in-stent restenosis, in non-AMI patients.**

|  | **ISR(+) (n=13)** | **ISR(-) (n=103)** | **P value** |
| --- | --- | --- | --- |
| **Demographic findings** |  |  |  |
| Age (years) | 66.4±11.2 | 60.2±9.2 | 0.027 |
| Sex (male ratio, %) | 84.6 | 86.4 | 0.860 |
| Clinical diagnosis (%) |  |  | 0.982 |
| Stable angina | 69.2 | 68.9 |  |
| Unstable angina | 30.8 | 31.1 |  |
| Hypertension (%) | 69.2 | 62.1 | 0.618 |
| Smoking (%) |  |  | 0.566 |
| Current smoker | 46.2 | 46.6 |  |
| Ex-smoker | 23.1 | 34.0 |  |
| Never smoker | 30.8 | 19.4 |  |
| Dyslipidaemia (%) | 46.2 | 43.7 | 0.866 |
| Previous MI (%) | 0 | 8.7 | 0.267 |
| **Laboratory findings** |  |  |  |
| Total cholesterol (mg/dl) | 181±43 | 182±38 | 0.921 |
| Triglyceride (mg/dl) | 161±81 | 157±90 | 0.869 |
| HDL-cholesterol (mg/dl) | 42±13 | 43±12 | 0.869 |
| LDL-cholesterol (mg/dl) | 97±30 | 103±31 | 0.507 |
| Serum creatinine (mg/dl) | 1.15±0.29 | 1.07±0.33 | 0.418 |
| hsCRP (mg/dl) | 0.59±0.77 | 0.90±2.06 | 0.610 |
| **Functional tests** |  |  |  |
| LV ejection fraction (%) | 63±6 | 58±11 | 0.109 |
| RWMA | 18.2 | 26.5 | 0.548 |
| Q wave in ECG | 30.8 | 21.6 | 0.455 |
| **Medication at follow-up** |  |  |  |
| Aspirin | 92.3 | 99.0 | 0.212 |
| Clopidogrel | 92.3 | 98.1 | 0.302 |
| ACE inhibitor or ARB | 84.6 | 67.0 | 0.339 |
| Statin | 92.3 | 86.4 | 0.550 |
