# Supplementary material for: Intravascular Ultrasound and Angiographic Predictors of In-Stent Restenosis of Chronic Total Occlusion Lesions
Source: PLoS One. 2015 Oct 14;10(10):e0140421. doi: 10.1371/journal.pone.0140421 (PMC4605613; doi:10.1371/journal.pone.0140421)
Supplement: S10 Table — (DOCX) [file pone.0140421.s012.docx]

**S10 Table. Angiographic, QCA and Post-PCI IVUS characteristics between patients with and without in-stent restenosis, in non-AMI patients.**

|  | **ISR(+) (n=13)** | **ISR(-) (n=103)** | **P** |
| --- | --- | --- | --- |
| **Angiographic Characteristics** |  |  |  |
| **Total number of stents inserted** | 2.1±0.8 | 1.7±0.7 | 0.071 |
| **Total stent length** | 58.4±24.8 | 44.8±19.1 | 0.022 |
| **Max. balloon expansion pressure** | 13.2±4.1 | 14.6±3.8 | 0.203 |
| **QCA Characteristics** |  |  |  |
| **Pre-PCI reference diameter (mm)** | 2.84±0.33 | 2.88±0.51 | 0.765 |
| **Post-PCI reference diameter (mm)** | 2.59±0.30 | 2.82±0.40 | 0.042 |
| **Post-PCI MLD (mm)** | 2.24±0.24 | 2.53±0.38 | 0.010 |
| **Post-PCI diameter stenosis (%)** | 11.5±10.1 | 10.46±7.0 | 0.725 |
| **F/U reference diameter (mm)** | 2.53±0.35 | 2.74±0.43 | 0.060 |
| **F/U MLD (mm)** | 0.89±0.90 | 2.26±0.46 | <0.001 |
| **F/U diameter stenosis (%)** | 70.8±25.8 | 17.8±12.4 | <0.001 |
| **Post-PCI IVUS Characteristics** |  |  |  |
| **Incomplete apposition** | 1 (7.7%) | 4 (4.1%) | 0.655 |
| **Proximal edge** | 1 | 3 |  |
| **Stent body** | 0 | 1 |  |
| **Distal edge** | 0 | 0 |  |
| **Tissue prolapse** | 1 (7.7%) | 14 (13.6%) | 0.550 |
| **Edge dissection** | 0 (0.0%) | 1 (1.0%) | 1.000 |
| **Proximal edge** | 0 | 0 |  |
| **Distal edge** | 0 | 1 |  |
| **MSA (mm^2^)** | 3.29±0.60 | 4.46±1.51 | <0.001 |
| **EEMA at MSA site (mm^2^)** | 8.78±2.80 | 10.99±4.64 | 0.096 |
| **MSA/EEMA (%)** | 41.2±13.9 | 44.1±16.0 | 0.528 |
| **SER (%)** | 59.6±11.5 | 76.1±20.2 | 0.004 |
| **Nominal CSA of stent (mm^2^)** | 5.67±1.35 | 5.91±1.63 | 0.618 |
| **Plaque CSA behind stent (mm^2^)** | 5.49±2.69 | 6.53±3.82 | 0.341 |
| **Percentage area stenosis (%)** | 58.9±13.9 | 55.9±16.0 | 0.528 |
